# Optimizing Tailored Communications for Health Risk Assessment: A Randomized Factorial Experiment of the Effects of Expectancy Priming, Autonomy Support, and Exemplification

**DOI:** 10.2196/jmir.7613

**Published:** 2018-03-01

**Authors:** Carmina G Valle, Tara L Queen, Barbara A Martin, Kurt M Ribisl, Deborah K Mayer, Deborah F Tate

**Affiliations:** ^1^ Department of Nutrition Gillings School of Global Public Health University of North Carolina at Chapel Hill Chapel Hill, NC United States; ^2^ Lineberger Comprehensive Cancer Center University of North Carolina at Chapel Hill Chapel Hill, NC United States; ^3^ Department of Health Behavior Gillings School of Global Public Health University of North Carolina at Chapel Hill Chapel Hill, NC United States; ^4^ School of Nursing University of North Carolina at Chapel Hill Chapel Hill, NC United States

**Keywords:** health communication, feedback, eHealth, health risk assessment, health behavior, intention, self-efficacy, personal autonomy

## Abstract

**Background:**

Health risk assessments with tailored feedback plus health education have been shown to be effective for promoting health behavior change. However, there is limited evidence to guide the development and delivery of online automated tailored feedback.

**Objective:**

The goal of this study was to optimize tailored feedback messages for an online health risk assessment to promote enhanced user engagement, self-efficacy, and behavioral intentions for engaging in healthy behaviors. We examined the effects of three theory-based message factors used in developing tailored feedback messages on levels of engagement, self-efficacy, and behavioral intentions.

**Methods:**

We conducted a randomized factorial experiment to test three different components of tailored feedback messages: tailored expectancy priming, autonomy support, and use of an exemplar. Individuals (N=1945) were recruited via Amazon Mechanical Turk and randomly assigned to one of eight different experimental conditions within one of four behavioral assessment and feedback modules (tobacco use, physical activity [PA], eating habits, and weight). Participants reported self-efficacy and behavioral intentions pre- and postcompletion of an online health behavior assessment with tailored feedback. Engagement and message perceptions were assessed at follow-up.

**Results:**

For the tobacco module, there was a significant main effect of the exemplar factor (*P*=.04); participants who received exemplar messages (mean 3.31, SE 0.060) rated their self-efficacy to quit tobacco higher than those who did not receive exemplar messages (mean 3.14, SE 0.057). There was a three-way interaction between the effect of message conditions on self-efficacy to quit tobacco (*P*=.02), such that messages with tailored priming and an exemplar had the greatest impact on self-efficacy to quit tobacco. Across PA, eating habits, and weight modules, there was a three-way interaction among conditions on self-efficacy (*P*=.048). The highest self-efficacy scores were reported among those who were in the standard priming condition and received both autonomy supportive and exemplar messages. In the PA module, autonomy supportive messages had a stronger effect on self-efficacy for PA in the standard priming condition. For PA, eating habits, and weight-related behaviors, the main effect of exemplar messages on behavioral intentions was in the hypothesized direction but did not reach statistical significance (*P*=.08). When comparing the main effects of different message conditions, there were no differences in engagement and message perceptions.

**Conclusions:**

Findings suggest that tailored feedback messages that use exemplars helped improve self-efficacy related to tobacco cessation, PA, eating habits, and weight control. Combining standard priming and autonomy supportive message components shows potential for optimizing tailored feedback for tobacco cessation and PA behaviors.

## Introduction

### Background

Health risk assessments plus feedback and additional educational approaches have been shown to be effective for supporting health behavior change [1]. Although many early health risk assessments were delivered via print [2], increasingly, these assessments are delivered via the Web. Web-based tailored interventions have demonstrated efficacy in promoting healthy behavior changes [3,4]. Online delivery of health risk assessments and tailored feedback present opportunities for greater reach and dissemination of effective health interventions that have the potential to lower costs, alleviate barriers to participation, and facilitate adherence to healthy behaviors. Although a large body of evidence supports the efficacy of tailored communications for promoting healthy behaviors (eg, [2,4-7]), there is limited evidence to guide the *optimal presentation of automated tailored feedback* on health behavior data to individuals [8]. Indeed, systematic reviews have called for researchers to provide enhanced descriptions of tailoring criteria and message design to optimize the use of Web-based tailored interventions to promote behavior change [4,9].

The Carolina Health Assessment and Resource Tool (CHART) is an online health behavior risk assessment tool developed at the University of North Carolina that includes various assessments of health behaviors related to chronic diseases that comprise the leading causes of premature death in the United States, along with evidence-based, theory-guided tailored feedback message libraries [2,4,10]. Individuals complete a baseline assessment on their current status in meeting the national recommendation for a specific health behavior (eg, physical activity [PA] and being tobacco-free), as well as other theory- and evidence-based psychosocial factors related to the health behaviors. Responses to this assessment are used within CHART to create a tailored feedback report (*personalized report*) based on an individuals’ reported current behavior, readiness to change, perceived barriers, and social support [10].

Message content and delivery format of tailored feedback reports, such as those offered by CHART, are critical components of online health messages that may affect an individual’s evaluation of a message, website use, and subsequent behavior change. To date, tailored health interventions have commonly used the major health behavior theories to guide message content (eg, social cognitive theory [SCT], transtheoretical model, and health belief model) [2,11,12]. Meta-analyses of tailored health behavior change interventions indicate that tailoring on more theoretical constructs (ie, 4-5 or more) in addition to behavior and demographics may improve the effectiveness of tailored interventions [2]. However, these theories contain many individual constructs, and there is a need to identify the specific message components of these tailored interventions in a systematic manner that will enhance understanding of the most effective message features and guide optimization and future testing of this set of features [13].

A growing literature has encouraged the use of the multiphase optimization strategy (MOST) framework to elucidate the active ingredients of interventions [13-15]. Thus, this study was designed to examine multiple message components (factors) of CHART personalized reports. The goals were to optimize the existing tailored feedback to promote self-efficacy and behavioral intentions for engaging in healthy behaviors and to enhance user engagement on the dimension of subjective experience. Guided by a recent systematic review and conceptual framework on engagement with digital behavior change interventions [16], our focus was on the experiential aspects of engagement, characterized by interest, affect, and attention (eg, self-report measures of perceptions of effectiveness, information quality, and attractiveness), and how engagement with the tailored feedback might be impacted by the content and delivery of the tailored feedback. Given that a single administration of a health risk assessment plus feedback, without additional intervention approaches, was unlikely to affect behavior change, we focused on self-efficacy and behavioral intentions. Both of these psychosocial constructs are key components of health behavior theories (eg, SCT and theory of planned behavior) and have been shown to be proximal determinants and predictors of behavior change [17,18]. Consistent with the screening phase of the MOST framework, we used a factorial design to allow for testing of the main effects on outcomes, as well as prespecified interactions. Our focus was on three specific message factors: expectancy priming, autonomy support, and exemplification, which are detailed below.

### Expectancy Priming

Individuals may vary on their tailoring-related expectancies, or the value or benefit that one may expect from tailored communications, and these expectancies are changeable [19]. Webb et al [20] demonstrated that individuals’ baseline expectancies about tailoring moderated the effect of personalized smoking cessation booklets on readiness to quit smoking, such that extensively personalized materials produced greater effects on readiness to quit among those with more positive expectancies about tailoring. A follow-up study showed that expectancy priming to manipulate tailoring-related expectancies (ie, making it clear that a message is either a standard one or personally tailored for that individual) can enhance the value or benefit that participants expect from either standard or tailored materials, improve ratings of message content, enhance readiness to change, and promote behavior change [19]. Thus, expectancy priming may influence the effect of tailored interventions such as feedback reports from health risk assessments. For this study, before receiving their tailored feedback report, participants were randomized to receive either a priming message explicitly stating that their feedback report was personally tailored for them, or they received a priming message stating that the feedback included a standard report.

### Autonomy Support

Behavioral interventions and tailored messages using self-determination theory (SDT) [21] as a guiding framework have demonstrated effectiveness for improving health behaviors such as PA [22], fruit and vegetable intake [23], and weight control [24]. SDT distinguishes between autonomous motivation and controlled motivation and posits that the *type of motivation*, rather than amount, is more influential on behavior [21,25]. Behaviors are autonomously motivated when actions result from conscious choice and are personally relevant, whereas controlled motivation involves engaging in a behavior because of perceived external pressures [26]. When individuals are autonomously motivated, behavior changes have been shown to be more effective and sustained [27]. Many health behavior interventions have focused on increasing autonomous motivation and in turn improved behavioral outcomes [22,28,29]. Often these interventions have encouraged autonomous motivation by incorporating autonomy supportive behavioral strategies based on SDT, including providing several options for change, supporting a sense of choice, eliciting an individual’s emotions, providing rationale for the importance of a behavior, and exploring the relevance of behaviors for an individual’s values and goals [30]. Thus, participants in this study were randomized to receive tailored feedback messages that were either autonomy supportive or used more directive language (ie, existing CHART feedback). On the basis of recommendations for enhancing autonomous motivation [26], the autonomy supportive messages offered a sense of choice or menu of options for change and encouraged participants to consider their own motivations and solutions to barriers, whereas the directive messages more explicitly told participants what to do.

### Exemplification

Self-efficacy, or confidence in one’s ability to take action or perform a behavior in the face of obstacles [31], is one of the most commonly targeted theoretical determinants in behavioral interventions and is central to multiple theories of behavior change (eg, SCT, transtheoretical model, and health belief model). Vicarious experiences and verbal persuasion are specific strategies relevant to health communication [32] and similar to exemplification used in media [33], which can be used to promote self-efficacy. Messages presented with an exemplar (eg, role model for the behavior) enable individuals to observe others perform an activity successfully, learn from vicarious experience, communicate positive outcomes of a behavior, and can encourage self-efficacy and in turn lead to behavior change [34-36]. Previous studies have shown that messages featuring exemplars, or role models, improve perceptions of self-efficacy and have the potential to enhance attractiveness of a message and promote positive behavior change [37]. In this study, participants were randomized to receive messages that featured an exemplar or did not use an exemplar.

Using manipulations of these three different message characteristics (expectancy priming, autonomy support, and exemplification), this study examined whether tailoring feedback messages using three different approaches improved self-efficacy and behavioral intentions to adhere to recommended health behaviors. As previous research has indicated that engagement with or subjective perceptions of tailored messages (eg, perceived message relevance) may mediate or explain the mechanism of tailoring effects [38-40], we also examined the effects of message factors on engagement, or subjective experiences related to attention, interest, and affect with tailored feedback messages. The overall goal was to inform the selection of the most effective messages for use in future CHART personalized reports and to expand the scientific basis for the optimal presentation of tailored feedback. We hypothesized that tailored expectancy priming, autonomy supportive messages, and messages with exemplars would be more effective for improving self-efficacy, behavioral intentions, and engagement compared with messages without these features.

## Methods

### Participants and Recruitment

Participants were recruited from January 2016 to February 2016 through Amazon Mechanical Turk (MTurk), a website where tasks are crowdsourced to employees, called workers, who receive compensation for completing Human Intelligence Tasks (HITs) [41]. MTurk has been used in a number of different research studies to collect a diversity of information such as health knowledge of ovarian cancer [42], ways to increase PA [43], and to measure body image [44] and the perceived harmfulness of tobacco products [45,46]. It is a useful tool for behavioral researchers because of its low cost, diverse worker population, and speed of data collection [41]. In MTurk, this study advertised the HIT as an opportunity to share opinions about a health survey and described the task as needing feedback about an online health website. Participants met the following initial eligibility criteria: aged 18 years or older, had Internet access, reside in the United States, able to communicate in English, and HIT approval rate (ie, percentage of worker’s completed HITs that have been approved by requestors) greater than or equal to 90%. Individuals were given US $1.25 for successful completion of the assignment, as detailed below. This study was reviewed and exempted by the institutional review board of the University of North Carolina at Chapel Hill (IRB #14-2873).

### Procedure and Design

We conducted pre- posttest experiments to test the effects of eight different message conditions on self-efficacy and intentions to engage in four different health behaviors.

**Figure 1:**
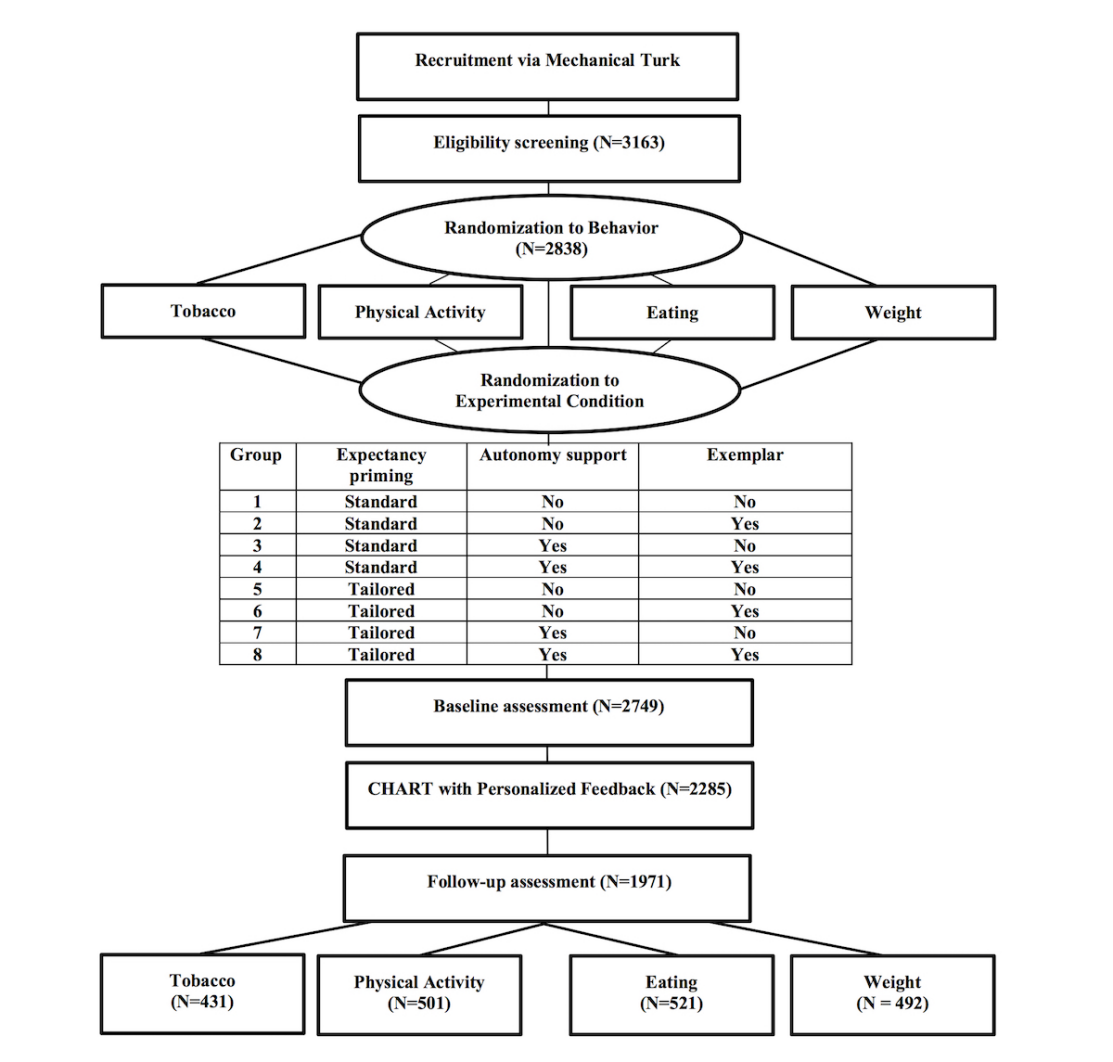
Flow of study participants in randomized 2x2x2 factorial experiment. CHART: Carolina Health Assessment and Resource Tool.

This study used a factorial design testing three experimental factors, with each factor having two levels of message characteristics (2x2x2): (1) expectancy priming before feedback delivery (standard vs tailored), (2) autonomy supportive messages (presence vs absence), and (3) use of exemplars (presence vs absence; Figure 1). We tested tailored feedback messages for four behavioral modules in CHART (tobacco use, PA, eating habits, and weight). For each of these four behaviors, the message characteristics were fully crossed (3 factors by 2 levels). Thus, for the independent variables of message condition, eight different cells were generated. Participants were randomized to one of the four behaviors (or 1 of 3, or 1 of 2, depending on eligibility, or assigned to 1 if only eligible for 1) and then subsequently randomly assigned, with equal probability, to one of eight experimental groups (Figure 1). The procedures from initial recruitment in MTurk through study completion are outlined below. In MTurk, the HIT indicated eligibility requirements to participate and provided a URL to begin the HIT. Participants were then directed through a series of online questionnaires and websites in the following order:

- This first questionnaire was an online screener with questions that asked individuals to report their gender and current smoking habits, PA behaviors, intake of fruits and vegetables, height, and weight. This screener identified participants that met additional eligibility criteria of not meeting national recommendations for at least one of four cancer prevention–related health behaviors: current tobacco use (smoker), PA (ie, less than 150 min of moderate exercise a week), fruit and vegetable consumption (ie, consuming less than 2 servings of fruit or 3 servings of vegetables daily), and weight status (ie, overweight or obese, body mass index ≥25).
- On the basis of eligibility, participants were randomized into one of the up to four health behaviors for which they were not meeting national recommendations. For instance, individuals who were not meeting national recommendations for all of the four behaviors were randomly assigned to one of the four modules. Individuals who were not meeting national recommendations for two behaviors (eg, PA and eating habits) were randomly assigned to one of these two modules. Respondents who did not meet the national recommendation for only one behavior were assigned to the corresponding module. Within each behavior, individuals were randomly assigned to one of eight different message conditions (Figure 1) based on three factors: expectancy priming (standard or tailored), autonomy support (yes or no), and exemplar (yes or no).
- Upon randomization, participants completed an online baseline questionnaire related to the specific behavioral module, which assessed self-efficacy and behavioral intentions. Participants received a unique user password after completing the questionnaire.
- Next, participants were directed to the CHART website [47], where they entered their password and completed an assessment questionnaire related to their assigned health behavior and a demographics questionnaire. Questions included standard items used in the CHART assessments (ie, current health behavior, readiness to change, barriers to engaging in the specific health behavior, and social support).
- Upon completion of the CHART assessment, participants received a personalized report that was tailored-based on preexisting tailoring variables programmed in CHART (current health behavior, readiness to change, barriers, and social support) and included messages with features consistent with one of the eight randomized conditions.
- Participants were instructed to read through the report and to click on a link that directed them to a password-protected final online questionnaire that asked about their opinions on the personalized report.
- Once participants completed the final questionnaire, they received a HIT completion code. After entering the completion code on the MTurk website, participants received US $1.25 for their time. Mean study completion time was 15.6 min (standard deviation [SD] 9.9).

Data collection for this study was completed in four cohorts.

### Experimental Conditions

Multimedia Appendix 1 provides examples of the message text in the various experimental conditions delivered through the personalized reports, which are detailed below.

#### Expectancy Priming

In the introduction to the personalized report, participants were randomized to receive either a standard priming or tailored priming message that described the contents of the report. The landing page used common graphics and language to direct individuals to click on a link to access their personalized report. The description of the personalized report differed between conditions and was adapted from previous research [20] (Multimedia Appendix 1). The standard priming condition indicated that the report was based on research that may help people meet recommendations. Language in the tailored priming condition stressed that the report was tailored especially for the individual and designed to meet their unique needs.

#### Autonomy Support

Participants were randomized to receive tailored feedback that consisted of either messages designed to be autonomy supportive or messages without a focus on autonomy support (standard existing CHART messages or exemplar messages). Tailored feedback in the autonomy supportive condition used language that was less directive and encouraged individuals to consider their own preferences and options. Original CHART-tailored feedback messages were revised and rephrased to ask more open-ended questions with the goal of promoting autonomy and choice (Multimedia Appendix 1). For instance, instead of directing individuals to “Fit weighing into your daily routine by stepping on the scale every morning when you get up,” the autonomy supportive messages asked, “How can you fit weighing into your daily routine? How about stepping on the scale every morning when you get up?”

#### Exemplar

In the exemplar condition, participants received messages that used gender-matched descriptions of a man (Bill) or woman (Rachel) who had a similar behavioral profile and had successfully made changes to meet the recommended behavioral goal. Tailored feedback in the original CHART message library was adapted to include Bill or Rachel as a role model for working toward improving their behaviors. For instance, an original CHART message read as follows: “Changing what you eat is not always easy. But, you can do it! Start with a goal you know you can reach. Small changes, like swapping sweetened drinks for water, can make a big difference to your health.” This same message was revised to include a role model for the behavior: “Changing what you eat is not always easy. But, you can do it! Like you, Rachel [Bill] had challenges that were getting in the way of her [his] healthy eating. She [He] started with a goal she [he] knew she [he] could reach and found that small changes, like swapping cookies for fruit, made a big difference to her [his] health.”

#### Autonomy Support x Exemplar

For participants that were randomized to this condition, tailored feedback combined messages that were autonomy supportive and included an exemplar (Multimedia Appendix 1). This was operationalized by using messages that were nondirective and asked questions that encouraged reflection, while also including Bill or Rachel as a role model exemplifying positive behaviors. For example, a standard tailored message regarding weight read as follows: “Try to fit veggies into every meal! Eating vegetables, especially those that are brightly colored, may help protect against heart disease and stroke.” This same message was revised to the following: “Have you thought about trying to fit veggies into every meal? Rachel [Bill] decided to eat more vegetables, especially brightly colored ones, since they can help protect her [him] against heart disease and stroke.”

### Measures

#### Primary Outcomes

*Self-efficacy* was assessed at baseline and after receipt of the personalized report using a single item adapted from previous studies [48,49] that asked participants “How confident are you that you can...” (1) Quit smoking or stop using smokeless tobacco products?, (2) Get the recommended amount of PA each week?, (3) Eat at least (5 for women, 5½ for men) cups of fruit and vegetables each day?, or (4) Control your weight? Responses ranged from 1 (*not at all confident*) to 5 (*extremely confident*).

*Behavioral intentions* were measured at baseline and following receipt of the personalized report with two (tobacco, weight) or four items (eating habits, PA) on a 7-point scale from *1 (strongly disagree) to 7 (strongly agree).* Items were adapted from previous measures [50-53] and asked participants to indicate the extent to which they agree or disagree with statements about their intentions to engage in a health behavior goal over the next month (eg, I intend to exercise regularly over the next month. I will try to exercise regularly over the next month.). Measures for all modules used the stems “I intend to...” and “I will try to...,” with the behavioral goals matching those appearing in the specific modules (ie, quit smoking and aim for a healthy weight). For the PA and eating habits measures, four items were used to assess behavioral intentions related to two behavioral goals (ie, exercise regularly, get at least 150 min of PA each week, eat at least two cups of fruits each day, and eat at least three cups of vegetables each day). Items were averaged for each behavior (Cronbach alpha=.92-.96).

#### Engagement and Perceptions of Personalized Report Messages

*Perceived message relevance*, which has been shown to be related to tailoring [6] and a mediator of behavior change [38,39], was measured with two items adapted from previous studies of tailored messages [38-40]. Participants were asked to rate how strongly they disagree or agree with the following statements: (1) “The information in the personalized report seemed to be written personally for me” and (2) “The information in the personalized report applied to my life.” Responses were on a 5-point scale from 1 (*strongly disagree*) to 5 (*strongly agree*) and were averaged across the two items (Cronbach alpha=.80).

*Perceived informativeness* was assessed using a 2-item scale adapted from Cho and Boster [54] that asked participants to rate their agreement with statements on a 5-point scale. Statements included (1) “The personalized report was informative” and (2) “I learned something from the personalized report,” and responses ranged from 1 (*strongly disagree*) to 5 (*strongly agree*). Items were averaged (Cronbach alpha=.84).

With respect to *perceived message quality*, participants were asked about their perceptions of the quality of the personalized report using a 5-item perceived message quality scale [54]. Items included statements such as “The personalized report was persuasive” and “I feel that the personalized report was convincing.” Response options ranged from 1 (*strongly disagree*) to 5 (*strongly agree*) and were averaged across the 5 items (Cronbach alpha=.93).

*Perceived trustworthiness* was assessed with one item [38,39]: “I believed the information in the personalized report.” Responses were on a 5-point scale from 1 (*strongly disagree*) to 5 (*strongly agree*).

To assess *perceived attractiveness*, participants were asked 1 item [40] on a 7-point scale (1=*very much* to 7=*not at all)*: “How attractive did you find the personalized report?”

For assessing *perceived message effectiveness*, a 3-item scale, adapted from Jensen et al [40] was used to ask participants about the persuasiveness of the personalized report. Questions asked (1) “Was the personalized report convincing?”; (2) Would people your age who smoke (who are not exercising regularly, who are not eating a healthy diet, and who are not at a healthy weight) be more likely to quit (to exercise regularly, to eat a healthier diet, and to aim for a healthy weight) after reading the personalized report?”; (3) “Would the personalized report be helpful in convincing your friends to quit smoking (to exercise regularly, to eat a healthy diet, and to aim for a healthy weight)?” Responses options ranged from 1 (*definitely no)* to 4 (*definitely yes*) and were averaged (Cronbach alpha=.89).

#### Engagement With Health Assessment Website

We adapted the 9-item Website Evaluation Questionnaire [55], originally developed to measure self-reported engagement, to ask participants about their evaluation of the overall CHART website. Responses were on a 5-point scale (1=*strongly disagree* to 5=*strongly agree*) and were averaged across three different items to derive three subscales. *Perceptions of personal relevance* assessed the degree to which participants felt the website was tailored (eg, “The information and advice provided by the website was appropriate for me”; Cronbach alpha=.85). The *perceptions of self-assessment and goal setting* subscale assessed the degree to which participants felt that the website helped them to reflect on their current behaviors and set goals (eg, “The website helped me to plan”; Cronbach alpha=.86), whereas the *engagement* subscale assessed the degree to which participants felt the website was attractive and enjoyable to use (eg, “The website was engaging”; Cronbach alpha=.89.).

For *satisfaction*, a single item asked participants the following: “How do you assess your participation in the online health assessment website in general?” Response options were on a 5-point scale and included 1 (*poor*), 2 (*average*), 3 (*good*), 4 (*very good)*, and 5 (*excellent*).

#### Demographic Characteristics

Participants reported age, sex, race, ethnicity, educational attainment, marital status, annual income, employment, and health insurance status. Data were collected through the CHART demographics module.

### Statistical Analyses

Data were examined for outliers and distributions. Given that the distribution of data relating to the tobacco module was markedly different from the other behaviors (eating habits, PA, and weight management), we analyzed the data related to tobacco separately from the other behaviors, whereas data on the three other behaviors were combined. In examining data, the distribution of the primary outcome variable (behavioral intentions) was similar across the PA, eating behavior, and weight modules, whereas it differed for the tobacco module. Consistent with a previous approach used to analyze data related to CHART [10], we collapsed data across the PA, eating behavior, and weight modules. Furthermore, as the tobacco module focused on an addictive behavior with a recommendation to quit and the other three behavioral modules were similar with respect to recommendations promoting adoption of behaviors, we anticipated that the messages might have similar effects across the three nontobacco behaviors.

We conducted multivariate analysis of variance to evaluate the main effect of each condition (ie, difference between mean response at one level of factor and mean response at other level, collapsing over the levels of all remaining factors) and interactions between conditions on our primary outcomes of interest (behavioral intentions and self-efficacy). Each model included the three experimental conditions, two-way and three-way interaction terms (expectancy priming x autonomy support, expectancy priming x exemplar, autonomy support x exemplar, and expectancy priming x autonomy support x exemplar), an intercept, and the grand mean-centered baseline measure of the outcome of interest as a covariate. 

For models related to the health behaviors other than tobacco, the assigned health behavior module was also included as a covariate. Estimated marginal means based on models are reported. We used a similar approach to examine the effect of experimental conditions on measures of engagement and message perceptions. As these measures were collected only in the follow-up questionnaire, analyses did not control for a baseline measure. All analyses were conducted using Statistical Package for the Social Sciences (SPSS) version 23.0 (IBM Corp).

## Results

### Participants

Of 3163 respondents in MTurk, 2838 completed the online screener and were randomized to 1 of 32 possible conditions (8 conditions across 4 behaviors). Among 2749 individuals who completed the baseline questionnaire, 2285 proceeded to take the CHART assessment. Upon reviewing their personalized reports, 1971 participants completed the follow-up questionnaire. Due to an error with skip patterns that resulted in missing responses related to self-efficacy, 155 participants in the tobacco module were excluded from analyses. An additional 26 participants were excluded because their website activity indicated cases with duplicate IDs and unpaired assessments because of technical issues, which resulted in 1945 participants used in analyses. Figure 1 and Table 1 show the number of participants analyzed by health behavior module and condition, respectively. Characteristics of the 1945 participants are summarized in Table 2. Participants (N=1945) were on average aged 36 years, with the majority being female (54.6%, 1062/1945), married (54.6%, 1062/1945), and college graduates (52.4%, 1019/1945).

### Effects on Primary Outcomes

#### Self-Efficacy

For tobacco-related self-efficacy, analyses revealed a significant main effect of the exemplar condition, *F*_1,266_=4.157, *P*=.04, η^2^=0.015. Participants who received exemplar messages (mean 3.31, SE 0.060) rated their self-efficacy to quit tobacco higher than those who did not receive exemplar messages (mean 3.14, SE 0.057). Expectancy priming (*F*_1,266_=0.836, *P*=.36) and autonomy support (*F*_1,266_=0.019, *P*=.89) conditions did not have main effects on tobacco-related self-efficacy.

**Figure 2:**
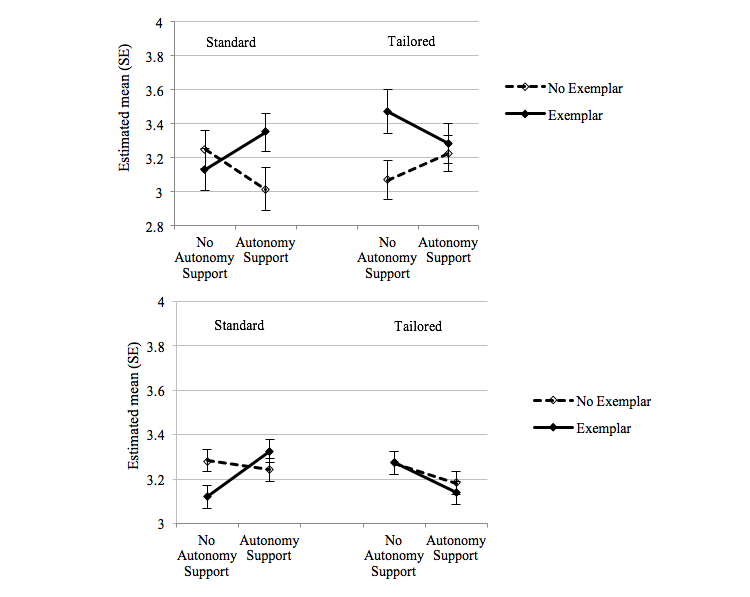
Estimated means (SE) for self-efficacy at follow-up as a function of three-way interaction of expectancy priming, autonomy support, and exemplar conditions. Error bars are SEs of the means. Higher scores represent higher self-efficacy. Tobacco (top): three-way interaction effect (*P*=.02) of autonomy support and exemplar conditions on self-efficacy to quit smoking, by priming condition. Physical activity, eating habits, weight (bottom): three-way interaction effect (*P*=.048) of autonomy support and exemplar conditions on self-efficacy to engage in physical activity, eating habits, and weight management behaviors, by priming condition.

The three-way interaction between the conditions was statistically significant (*F*_1,266_=5.807, *P*=.02, η^2^=0.021) and is illustrated in Figure 2. The strength of the effect of the exemplar condition was moderated by the other conditions. For those in the tailored priming condition, mean self-efficacy was highest among those who received the exemplar message with no autonomy support (mean 3.47, SE 0.129). The next highest self-efficacy scores were among those in the standard priming condition, who received both the exemplar and autonomy supportive messages (mean 3.35, SE 0.111).

Results on the health behaviors other than tobacco showed no significant main effects of the three experimental conditions on self-efficacy at follow-up (priming: *F*_1,1501_=0.518, *P*=.47; autonomy support: *F*_1,1501_=0.165, *P*=.685; and exemplar: *F*_1,1501_=0.695, *P*=.41). 

**Table 1:** Experimental conditions and cell sizes by health behavior module.

Group	Experimental condition			Health behavior module (N)			
	Tailored expectancy priming	Autonomy support	Exemplar	Tobacco	Physical activity	Eating habits	Weight
1	No	No	No	56	71	62	69
2	No	No	Yes	57	62	62	61
3	No	Yes	No	42	64	67	63
4	No	Yes	Yes	57	66	63	60
5	Yes	No	No	58	59	78	61
6	Yes	No	Yes	52	59	62	57
7	Yes	Yes	No	54	59	61	63
8	Yes	Yes	Yes	55	61	66	58

**Table 2:** Characteristics of participants (N=1945) in experiments assessing Carolina Health Assessment and Resource Tool (CHART) personalized reports.

Characteristic		Value
Age in years, mean (SD)		36.22 (11.01)
**Sex, n (%)**		
	Female	1062 (54.60)
	Male	883 (45.40)
**Race^a^, n (%)**		
	Non-Hispanic white	1675 (86.12)
	African American	148 (7.61)
	Asian	134 (6.89)
	American Indian or native American	41 (2.11)
	Pacific Islander	4 (0.21)
	Other	21 (1.08)
**Ethnicity, n (%)**		
	Hispanic	128 (6.58)
**Marital status, n (%)**		
	Married or living as married	1062 (54.60)
	Not married	883 (45.40)
**Education, n (%)**		
	≤High school	230 (11.83)
	Some college or technical school	696 (35.78)
	≥College graduate	1019 (52.39)
**Annual income (USD), n (%)**		
	<$35,000	792 (40.72)
	$35,000 to <$75,000	727 (37.38)
	≥$75,000	379 (19.49)
**Employment status, n (%)**		
	Employed	1385 (71.21)
	Not employed	552 (28.38)
Have health insurance, n (%)		1564 (80.41)

^a^Participants could choose all that apply.

The two-way interaction effect of priming and autonomy support conditions was significant (*F*_1,1501_=7.028, *P*=.008, η^2^=0.005), such that the effect of autonomy support was higher within the standard priming condition (mean 3.28, SE 0.036) than the tailored priming condition (mean 3.16, SE 0.037). As with self-efficacy related to tobacco, the three-way interaction among the conditions had a significant effect on self-efficacy related to the other health behaviors (*F*_1,1501_=3.925, *P*=.048, η^2^=0.003).

Figure 2 shows the effect of autonomy support and exemplar conditions by priming condition. The exemplar condition increased the effect of autonomy support within the standard priming condition, such that the highest self-efficacy scores were reported among those who were in the standard priming condition and received both autonomy and exemplar messages (mean 3.33, SE 0.052). Within the tailored priming condition, mean self-efficacy scores were lowest among those in the autonomy support condition, with (mean 3.14, SE 0.052) or without an exemplar message (mean 3.18, SE 0.053).

**Figure 3:**
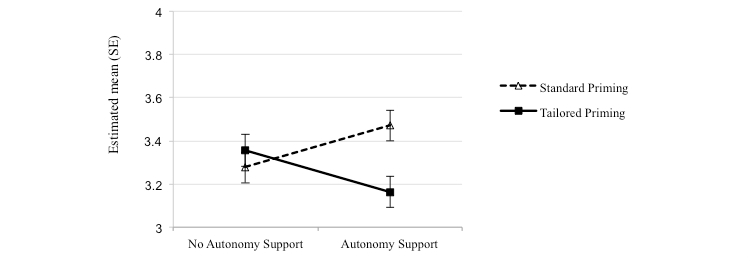
Estimated means (SE) for self-efficacy for physical activity at follow-up as a function of two-way interaction of expectancy priming and autonomy support. Error bars are SEs of the means. Higher scores represent higher self-efficacy.

In analyses of self-efficacy by individual health behaviors, we found that the significant interaction effect of expectancy priming and autonomous support was specific to those within the PA module (*F*_1,491_=7.185, *P*=.008, η^2^=0.014). Figure 3 illustrates the two-way interaction, such that autonomy support had stronger effects on self-efficacy for PA in the standard priming condition (mean 3.47, SE 0.070), whereas messages without autonomy support had stronger effects in the tailored priming condition (mean 3.36, SE 0.074).

**Figure 4:**
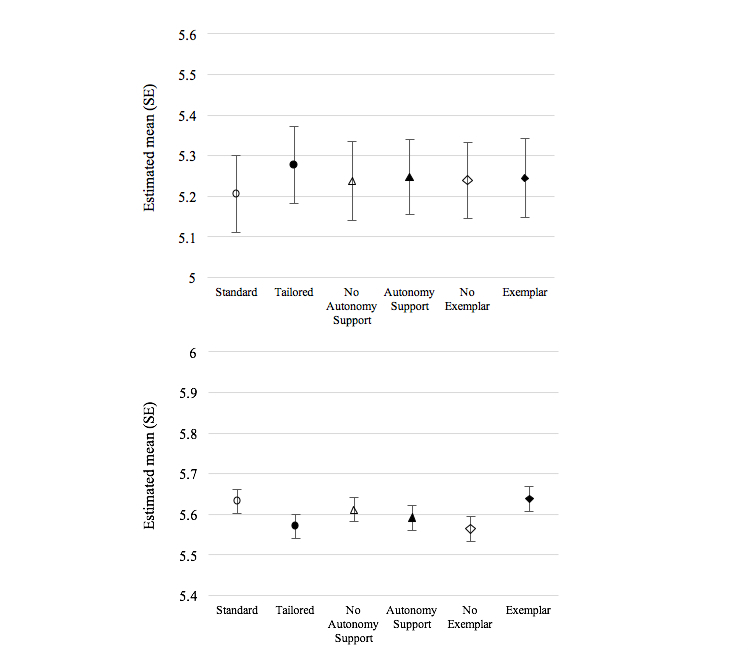
Estimated means (SE) for behavioral intentions at follow-up as a function of main effects of experimental conditions. Error bars are SEs of the means. Higher scores represent higher behavioral intentions. Tobacco (top): priming (*P*=.59), autonomy support (*P*=.94), and exemplar (*P*=.97) effects on behavioral intentions to quit smoking, controlling for baseline intention scores. Physical activity, eating habits, weight (bottom): priming (*P*=.15), autonomy support (*P*=.64), and exemplar (*P*=.08) effects on behavioral intentions to engage in other health behaviors, controlling for baseline intention scores.

#### Behavioral Intentions

Figure 4 shows the results for behavioral intentions to quit smoking. There were no significant main effects of the three conditions. Behavioral intentions among participants that received the standard priming message (mean 5.21, SE 0.095) did not differ from those who received the tailored priming message (mean 5.28, SE 0.095; *F*_1,267_=0.292, *P*=.59). Similarly, there was no main effect for autonomy support (mean 5.25, SE 0.093) versus no autonomy support (mean 5.24, SE 0.097; *F*_1,267_=0.005, *P*=.94), or the exemplar (mean 5.24, SE 0.097) versus no exemplar (mean 5.24, SE 0.094; *F*_1,267_=0.001, *P*=.97). No significant interactions were found among the experimental conditions.

For the other health behaviors (Figure 4), the main effect of exemplar messages on behavioral intentions was in the hypothesized direction but did not reach statistical significance (*F*_1,1503_=3.026, *P*=.08). Mean behavioral intention scores were 5.64 (SE 0.031) for those who received exemplar messages and 5.56 (SE 0.030) for those who did not. There were no main effects of priming or autonomy support. Mean behavioral intention scores were similar between standard priming (mean 5.63, SE 0.030) and tailored priming conditions (mean 5.57, SE 0.030; *F*_1,1503_=2.06, *P*=.15) and between the autonomy support (mean 5.59, SE 0.030) and no autonomy support (mean 5.61, SE 0.030; *F*_1,1503_=0.223, *P*=.64) conditions. There were no significant interactions between any of the experimental conditions.

**Table 3:** Engagement and perceptions of the Carolina Health Assessment and Resource Tool (CHART) personalized report and website at follow-up (tobacco module).

Scale	Scale range	Tobacco, mean (SD)					
		Standard priming (N=200)	Tailored priming (N=211)	No autonomy support (N=213)	Autonomy support (N=198)	No exemplar (N=202)	Exemplar (N=209)
Perceived message relevance	1-5	3.58 (0.91)	3.64 (0.90)	3.59 (0.92)	3.62 (0.90)	3.59 (0.89)	3.62 (0.93)
Perceived informativeness	1-5	3.69 (0.88)	3.74 (0.97)	3.72 (0.91)	3.71 (0.94)	3.71 (0.95)	3.73 (0.91)
Perceived quality	1-5	3.63 (0.87)	3.63 (0.92)	3.64 (0.90)	3.62 (0.88)	3.63 (0.88)	3.63 (0.90)
Perceived trustworthiness	1-5	3.94 (0.85)	4.01 (0.89)	3.97 (0.91)	3.99 (0.82)	3.93 (0.89)	4.02 (0.85)
Perceived attractiveness	1-7	3.97 (1.66)	3.89 (1.76)	3.80 (1.74)	4.06 (1.68)	3.95 (1.71)	3.91 (1.72)
Perceived message effectiveness	1-4	2.73 (0.63)	2.77 (0.65)	2.74 (0.62)	2.75 (0.65)	2.72 (0.66)	2.78 (0.62)
Perceptions of personal relevance	1-5	3.69 (0.76)	3.70 (0.83)	3.66 (0.82)	3.73 (0.78)	3.68 (0.80)	3.71 (0.79)
Perceptions of self-assessment and goal setting	1-5	3.47 (0.91)	3.53 (0.93)	3.47 (0.90)	3.54 (0.93)	3.45 (0.91)	3.56 (0.92)
Engagement	1-5	3.57 (0.89)	3.62 (0.96)	3.58 (0.90)	3.62 (0.94)	3.48 (0.96)	3.72 (0.87)
Participation in CHART^a^	1-5	3.68 (0.91)	3.67 (0.93)	3.68 (0.89)	3.67 (0.95)	3.57 (0.96)	3.78 (0.87)

^a^CHART: Carolina Health Assessment and Resource Tool.

**Table 4:** Engagement and perceptions of the Carolina Health Assessment and Resource Tool (CHART) personalized report and website at follow-up (physical activity, eating behaviors, and weight modules).

Scale	Scale range	Physical activity, eating behaviors, and weight, mean (SD)					
		Standard priming (N=744)	Tailored priming (N=715)	No autonomy support (N=743)	Autonomy support (N=716)	No exemplar (N=744)	Exemplar (N=715)
Perceived message relevance	1-5	3.57 (0.88)	3.56 (0.92)	3.59 (0.89)	3.54 (0.89)	3.59 (0.89)	3.55 (0.92)
Perceived informativeness	1-5	3.75 (0.94)	3.72 (0.94)	3.70 (0.96)	3.76 (0.92)	3.73 (0.94)	3.74 (0.94)
Perceived quality	1-5	3.63 (0.88)	3.62 (0.90)	3.61 (0.89)	3.64 (0.88)	3.63 (0.88)	3.61 (0.89)
Perceived trustworthiness	1-5	3.96 (0.85)	3.95 (0.86)	3.96 (0.88)	3.95 (0.83)	3.93 (0.88)	3.97 (0.83)
Perceived attractiveness	1-7	3.89 (1.69)	3.89 (1.68)	3.93 (1.70)	3.85 (1.67)	3.90 (1.72)	3.88 (1.72)
Perceived message effectiveness	1-4	2.83 (0.61)	2.81 (0.62)	2.82 (0.62)	2.82 (0.61)	2.81 (0.62)	2.83 (0.62)
Perceptions of personal relevance	1-5	3.64 (0.81)	3.65 (0.84)	3.64 (0.83)	3.66 (0.82)	3.67 (0.80)	3.62 (0.85)
Perceptions of self-assessment and goal setting	1-5	3.54 (0.89)	3.55 (0.89)	3.52 (0.89)	3.57 (0.90)	3.57 (0.86)	3.52 (0.93)
Engagement	1-5	3.65 (0.89)	3.63 (0.88)	3.64 (0.88)	3.64 (0.90)	3.65 (0.89)	3.63 (0.89)
Participation in CHART^a^	1-5	3.63 (0.96)	3.67 (0.93)	3.65 (0.93)	3.65 (0.97)	3.66 (0.95)	3.63 (0.95)

^a^CHART: Carolina Health Assessment and Resource Tool.

### Effects on User Engagement and Message Perceptions

Tables 3 and 4 show the mean scores related to engagement and perceptions of the personalized report and CHART website by main effects of the experimental conditions. Overall, participants reported positive perceptions of the tailored feedback reports and CHART website. Mean ratings of the personalized report regarding perceived message relevance, informativeness, message quality, and trustworthiness (1=*strongly disagree* to 5=*strongly agree*) ranged from 3.58 (SD 0.91) to 4.02 (SD 0.85) for the tobacco module (Table 3) and from 3.54 (SD 0.89) to 3.97 (SD 0.83) for the nontobacco modules (Table 4). Participants across both the tobacco-related and nontobacco modules reported average scores for attractiveness of the personalized reports (1=*very much* to 7=*not at all*), ranging from 3.80 (SD 1.74) to 4.06 (SD 1.68) and 3.85 (SD 1.67) to 3.93 (SD 1.70), respectively.

Message effectiveness of personalized reports (1=*definitely no* to 4=*definitely yes*) was rated more positively for both the tobacco module and other behaviors. Evaluations of the overall CHART website (1=*strongly disagree* to 5=*strongly agree*) were generally positive across all three subscales (personal relevance, perceptions of self-assessment and goal setting, and engagement). Scores ranged from 3.45 (SD 0.91) to 3.78 (SD 0.87) for the tobacco modules (Table 3) and 3.52 (SD 0.93) to 3.67 (SD 0.80) for the nontobacco modules (Table 4). Satisfaction ratings regarding participation in CHART fell between *good* to *very good* across all modules. There were no significant differences in engagement and perceptions among groups.

## Discussion

### Principal Findings

Findings from this randomized factorial experiment showed that tailored priming before presentation of a tailored feedback report, with use of an exemplar to model smoking cessation behavior, produced the largest effect on self-efficacy in the tobacco module. For the other three modules (PA, eating habits, and weight), self-efficacy was highest among those who received standard priming of the feedback and messages offering both autonomy support and an exemplar. Messages featuring tailored expectancy priming, autonomy support, or exemplars did not improve behavioral intentions to engage in healthy behaviors or result in differences in engagement and message perceptions. These empirical findings on various theory-driven messages delivered in response to an online health risk assessment contribute to the relatively sparse literature guiding the optimal presentation of online tailored feedback to individuals. Overall, results of this study suggest that using exemplars in tailored feedback messages has the potential to improve self-efficacy in the early phases of behavior change interventions. Furthermore, the addition of standard priming messages before presentation of feedback, along with autonomy supportive messages, could help optimize message effects on self-efficacy.

### Comparison With Prior Work

#### Self-Efficacy

##### Exemplars

In this study, using an exemplar showed potential for improving self-efficacy across all of the behaviors (tobacco, PA, eating habits, and weight management). Among participants in the tobacco module, messages with exemplars produced the highest self-efficacy scores. Strecher et al [36] previously demonstrated that high-depth tailored (ie, tailored to several characteristics beyond name and gender) success stories delivered through a Web-based smoking cessation program were effective for improving smoking abstinence at 6 months. Similarly, Sarge and Knobloch-Westerwick [56] showed that using exemplars in an online health article that modeled successful weight loss behavior improved weight loss self-efficacy. The positive effects on self-efficacy observed among those receiving exemplar messages are consistent with SCT and strategies such as observational learning, vicarious experience, and verbal persuasion that are theorized to promote self-efficacy [32,34,35].

##### Expectancy Priming

Interestingly, the highest self-efficacy scores among participants in the tobacco module were observed in those who received exemplar messages along with tailored priming, whereas the next highest were among those receiving exemplar messages with standard priming and autonomy supportive messages. Likewise, for the other health behavior modules (PA, eating habits, and weight), messages that included standard priming with autonomy supportive and exemplar messages resulted in the highest self-efficacy scores. The potentially positive effects of priming are consistent with work by Webb et al [19], which showed that pretreatment expectancy priming (both standard and tailored) of smoking cessation materials resulted in improvements in readiness to quit smoking and smoking-related knowledge, although not self-efficacy. Another study showed that patients who were primed with physician advice before receiving printed health education materials were more likely than those who did not receive physician advice, to report changes in diet and PA and attempt to quit smoking [57]. Our findings suggest that the inclusion of expectancy priming, such as making it clear that a message is personally tailored for that individual, before delivery of personalized feedback may improve the effects of tailored messages in the context of online health risk assessment tools.

##### Autonomy Support

The interaction between the effects of standard priming and autonomy supportive messages on self-efficacy (ie, the effect of autonomy support was higher in the standard priming condition) demonstrates potential for further study. It is unclear why self-efficacy would be higher among those receiving the standard priming and autonomy supportive messages. It is possible that the standard priming influenced individuals’ positive expectancies related to standard health messages, and viewing subsequent autonomy supportive messages matched expectations that the feedback report would be more general in nature, as the messages offered various options and encouraged individuals to reflect on their own preferences. Previous research has shown that among participants receiving three tailored newsletters aimed at improving autonomous motivation, those who preferred and received more autonomy supportive communication increased their fruit and vegetable intake relative to those who received tailored newsletters not focused on autonomous motivation [12]. Few research studies have examined the effectiveness of tailoring online health communication messages based on individuals’ need or preference for autonomy [58]. Future work examining the mechanisms of interaction between expectancy priming and messages using either autonomy supportive or exemplar messages appear warranted. In particular, identifying and assessing preferences for autonomy supportive communication, or more narrative forms that include behavioral models, may help improve the development of more personally relevant messaging in tailored feedback communications. Given the scarcity of research that has evaluated online health communications that tailor message framing to match an individuals’ need for autonomy and other information processing styles [58], there is a need to further elucidate the effectiveness of tailoring messages based on autonomy supportive preferences. Studies that examine the effects of tailoring on need for autonomy alone or in combination with other psychosocial constructs could advance our understanding of the potential to improve the effectiveness of online health communications that are tailored to individuals’ information processing preferences.

#### Behavioral Intentions

Although we expected to observe improvements in behavioral intentions as a result of the various message enhancements, this was not borne out in our findings. In the nontobacco modules (PA, eating habits, and weight), there was a nonsignificant trend for messages with exemplars to improve behavioral intentions to engage in the behaviors. This lack of effect on behavioral intentions is similar to findings from other message testing studies that have sought to improve intentions for various health behaviors using a one-time delivery of targeted or framed messages [59-61]. In the context of a computer-tailored nutrition intervention, Oenema et al [62] showed that the tailored intervention improved intentions to change vegetable consumption relative to generic nutrition information and no-information control groups. This effect was mediated by perceived message relevance and perceived individualization. Given that the existing CHART messages were already tailored to individuals’ current behaviors and other psychosocial factors and aimed at improving behavioral intentions, it is possible there was a ceiling effect with little room for improvement beyond that produced by existing messages. Furthermore, the nonsignificant differences in behavioral intentions may be attributed to the lack of differences in perceived message relevance among the three message factors. Although behavioral intentions is a common construct in health behavior theories, studies highlight the gap between behavioral intention and subsequent behavior [63] and have shown that people’s expectations about what they will do are more predictive of subsequent behavior than their intentions [64]. Future studies of the effectiveness of tailored feedback messages might consider alternative outcome measures that have demonstrated better predictive validity of behavior, such as expectations, and examine the effects of multiple or frequent feedback reports delivered over time.

#### User Engagement and Message Perceptions

The comparability across the message factors with respect to perceptions and engagement with the personalized reports and CHART website suggests that the message conditions were equally appealing, relevant, and engaging. Previous studies have found that a variety of message perceptions (eg, relevance, persuasiveness, importance, and helpfulness) have mediated the relationship between tailored messaging and behaviors or behavioral intentions [38-40,62,65]. For example, perceived message relevance has been shown to be a mediator of the positive effects of tailored messaging on fruit and vegetable intake [38,39], vegetable intake intentions [62], and breast screening intentions [40], indicating the existence of significant relationships between the tailored materials and perceived message relevance.

In this study, we did not find such associations between the tailoring enhancements and message perceptions. Given that all experimental conditions provided tailored feedback messages, it is not surprising that there was little variation in perceptions and user engagement among the message factors. The relatively slight differences in phrasing of messages may not have been sufficient to influence various message perceptions as overall content and suggestions for making healthy behavior changes were consistent across conditions. The ratings for engagement with and perceptions of the CHART website indicate that participants found the overall health assessment website with feedback to be engaging and personally relevant. Participant ratings in this study were comparable with findings on self-reported engagement in a study that compared the effects of Internet-delivered assessment with and without tailored feedback versus generic information on self-management of bowel problems [55]. A recent systematic review provided an integrative definition of engagement with online behavior change interventions, which incorporates both subjective experiences and extent of usage, and offered a conceptual framework to guide measurement and evaluation of the relationship between engagement and intervention effectiveness [16]. Future research on tailored personalized feedback could incorporate both subjective and objective measurements of engagement to identify specific dimensions of engagement that have a greater influence on the effectiveness of health messages. Further elucidation of whether engagement may mediate or moderate the relationship between tailored feedback messages and intended behavioral outcomes is necessary.

### Limitations

While this study used an innovative strategy and factorial design to efficiently identify the most impactful message conditions to optimize tailored feedback reports, several limitations should be considered. Participants completed the pre- and postfeedback assessments during a single occasion, so the persistence of findings over time is unknown.

Although improving self-efficacy for behavior change and behavioral intentions may require more than a one-time administration of a health risk assessment with tailored feedback, our study was an experiment designed to test the effects of different message features on psychosocial factors and not an intervention for behavior change. Our findings may have implications for creating more impactful messages within the context of behavior change interventions. The sample recruited through MTurk was relatively homogeneous with respect to age, race, ethnicity, and education, which limits generalizability of our findings to a more diverse population. However, this recruitment approach facilitated rapid and efficient testing of multiple message conditions over a short period of time, and results are useful for generating hypotheses to be tested in future alternate populations that are harder and most costly to recruit. As all measures and outcomes were self-reported, over- or underreporting and responses influenced by social desirability may have biased our results, though presumably randomization would have evenly distributed any biased reporting across conditions.

Another limitation was the lack of an experimental condition without priming. Although this precluded us from evaluating the effects of any priming (whether standard or tailored) compared with none, we observed an interesting interaction effect, whereby combining standard priming with autonomy supportive messages resulted in higher self-efficacy scores. Finally, we observed relatively small effects of the message manipulations on our outcomes of interest. To minimize participant burden, we opted to use a single-item to assess self-efficacy, a limitation that may have led to insufficient variance to examine group differences. Although participants’ mean intentions and self-efficacy scores were relatively high at baseline, which possibly resulted in a ceiling effect and diminished our ability to observe associations, the large sample size was adequately powered to detect some differential effects among the message conditions.

### Conclusions

Overall, our study findings among MTurk workers suggest message characteristics that have the potential to enhance message impact on self-efficacy. In the context of an online health behavior assessment tool, the use of exemplars to convey tailored feedback may help promote improvements in self-efficacy related to tobacco cessation, PA, eating habits, and weight control. As findings among MTurk workers may not generalize to others who are seeking behavioral interventions, further evaluation of whether exemplars, priming, and autonomy supportive messages can enhance the impact of tailored feedback on cancer prevention–related behaviors among other populations is warranted.

## Abbreviations

CHART: Carolina Health Assessment and Resource Tool
HIT: Human Intelligence Task
MOST: multiphase optimization strategy
MTurk: Amazon Mechanical Turk
PA: physical activity
SCT: social cognitive theory
SDT: self-determination theory

## Appendix

Multimedia Appendix 1 Examples of experimental stimuli in personalized reports by condition.
