# Supplementary material for: Optimizing Tailored Communications for Health Risk Assessment: A Randomized Factorial Experiment of the Effects of Expectancy Priming, Autonomy Support, and Exemplification
Source: J Med Internet Res. 2018 Mar 1;20(3):e63. doi: 10.2196/jmir.7613 (PMC5856933; doi:10.2196/jmir.7613)
Supplement: Multimedia Appendix 1 [file jmir_v20i3e63_app1.pdf]

Appendix 1. Examples of experimental stimuli in personalized reports by condition.

Condition	Presence of condition[a]	
	No	Yes
Tailored expectancy priming	Congratulations on completing all the modules. Your report is based on information that research shows help people meet recommendations. You are receiving this report because it may help you meet recommendations.<br><br>Your report contains several different sections for you to review. Your CHART Summary is shown below, using the following key:<br><br>- you meet recommendations<br>- you can improve	Congratulations on completing all the modules. Your report has been tailored especially for you and is based on the information you provided. You are receiving a unique report that was designed to meet your unique needs.<br><br>Tailored information focuses your attention on the most important topics for you. Your time is not wasted reading information that does not apply to you. With your tailored report, you will only receive information that is relevant to you. Your CHART Summary is shown below, using the following key:<br><br>- you meet recommendations<br>- you can improve
Autonomy support	**Your Activity Level**<br><br>Give your activity a boost! Getting 150 minutes of physical activity can help you look your best and keep your body and mind healthy. Find an activity that you enjoy and fits your lifestyle. Take a walk with a friend during the day, or with a family member or pet after dinner, or be active with friends on the weekend.	**Your Activity Level**<br><br>What steps could you take to give your activity a boost? Did you know that being physically active for 150 minutes per week can help you look your best and keep your body and mind healthy? To start exercising more, one suggestion is to find an activity that you enjoy and fits your lifestyle. For example, could you take a walk with a coworker during the day or with a family member or pet after dinner? You could also try to be active with friends on the weekend.
Exemplar	**Your Tobacco Use**<br><br>It's time to quit! Quitting tobacco is one of the best things you can do for your health and the health of those around you. After just a few days without tobacco, you will start to feel better and save money. Over time, you will be less likely to have a heart attack or suffer many types of cancer. Commit to quit and tell your family, friends, and coworkers about your goal.	**Your Tobacco Use**<br><br>It’s time to quit! Quitting tobacco is one of the best things you can do for your health and the health of those around you. Like you, Bill used tobacco. After just a few days without tobacco, Bill started to feel better and saved money. Over time, Bill will be less likely to have a heart attack or suffer from many types of cancer. He committed to quitting and told his family, friends, and coworkers about his goal.
Autonomy support × Exemplar	**Your Tobacco Use**<br><br>It's time to quit! Quitting tobacco is one of the best things you can do for your health and the health of those around you. After just a few days without tobacco, you will start to feel better and save money. Over time, you will be less likely to have a heart attack or suffer many types of cancer. Commit to quit and tell your family, friends, and coworkers about your goal.	**Your Tobacco Use**<br><br>Have you thought about quitting tobacco? Did you know quitting tobacco is one of the best things you can do for your health and the health of those around you? Like you, Bill used tobacco. After just a few days without tobacco, Bill started to feel better and saved money. Over time, Bill will be less likely to have a heart attack or suffer many types of cancer. He committed to quitting and told his family, friends, and coworkers about your goal. What can you do to commit to quitting?

[a]Examples of feedback for conditions are presented for physical activity and tobacco.
